# Forest Cover and Geographic Distance Shape Ant Assemblages in the Southwestern Brazilian Amazon

**DOI:** 10.1002/ece3.72467

**Published:** 2025-11-12

**Authors:** Marília Maria Silva da Costa, Fernando Augusto Schmidt, Icaro Wilker, Chaim José Lasmar, Carla Rodrigues Ribas

**Affiliations:** ^1^ Programa de Pós‐Graduação Em Ecologia Aplicada, Departamento de Ecologia e Conservação Universidade Federal de Lavras Lavras Brazil; ^2^ Programa de Pós‐Graduação Em Ecologia, Instituto de Biologia Universidade Federal do Rio de Janeiro Rio de Janeiro Brazil; ^3^ Centro de Ciências Biológicas e da Natureza Universidade Federal do Acre Rio Branco Brazil; ^4^ Centro de Estudos Integrados da Biodiversidade Amazônica (CENBAM) Instituto Nacional de Pesquisas da Amazônia Manaus Brazil; ^5^ Instituto Nacional da Mata Atlântica Santa Teresa Brazil; ^6^ Departamento de Biologia, Programa de Pós‐Graduação Em Biodiversidade Universidade Federal Rural de Pernambuco Recife Brazil

**Keywords:** beta diversity, habitat‐use guilds, neotropical region, precipitation, species richness

## Abstract

Understanding the drivers affecting species richness and composition in local communities is crucial for discerning differences among communities in a specific region. In large regions, a variety of environmental conditions shape diversity, reflecting the peculiarities of each location, such as available resources and species interactions. While many species are associated with forest habitats, some can adapt to open environments, reflecting how environmental changes affect ant assemblages. The southwestern Brazilian Amazon stands out for significant contrasts in precipitation and forest cover, with the drier and deforested east compared to the wetter and forested west. Thus, we investigated how precipitation, forest cover and spatial distance influence ant species richness and composition. We conducted the study by selecting 16 sampling areas. We collected 365 species, of which 151 were classified based on their habitat use. We observed that increased forest cover is directly related to increased ant species richness, especially for forest specialists, highlighting the sensitivity of these species to forest habitat loss or gain. Additionally, we found that spatial distance between communities plays a significant role in explaining variance in ant species composition, surpassing the influence of forest cover. However, species composition is still influenced by variability in forest cover and precipitation, even when controlling for geographical distance. Our results provide important insights into ant diversity and composition in the Amazon. We emphasize the importance of considering the different responses of ants to environmental factors, using habitat‐use guilds, to guide effective biodiversity conservation measures.

## Graphical abstract

We investigated how forest cover, precipitation, and spatial distance influence ant species richness and composition in the southwestern Brazilian Amazon. We found that forest cover increases species richness, especially of forest specialists, while spatial distance is the main driver of variation in species composition. Our results highlight the importance of considering habitat‐use guilds and multiple environmental factors to support effective conservation strategies.

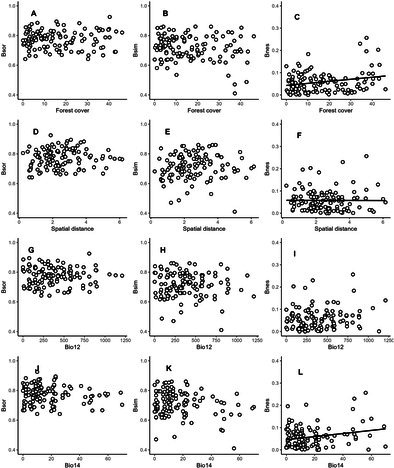

## 1 Introduction

Understanding the drivers of species richness and species composition among local communities within a region is a central goal of ecology (Blackburn and Gaston 2006; Gotelli et al. 2009; Martins et al. 2021; Jiménez‐Alfaro et al. 2023). Identifying areas with high species richness and understanding the factors that shape their distribution (Leach et al. 2016; Deacon et al. 2020; Arruda et al. 2020) offers important theoretical advances in explaining differences in species composition among communities. These findings also provide practical guidance for conservation strategies, particularly in the face of increasing habitat loss (Arantes et al. 2018; Macedo‐Reis et al. 2019; Pyles et al. 2020; Farneda et al. 2020).

While precipitation and temperature are widely recognized as primary drivers of diversity across a broad range of taxa (Gotelli et al. 2009; Prather et al. 2020; Korell et al. 2021), their influence is not always readily apparent, especially in studies considering limited climatic variation due to their reduced scale (Field et al. 2009). However, in extensive geographical regions, a broader array of environmental conditions exists, to which populations are exposed, influencing differences among local communities (Sites and Marshall 2004). Various factors, such as land cover, climatic variables, and geographical characteristics that vary across geographic extent, contribute to shaping species diversity on large spatial scales (Hawkins et al. 2003; Solar et al. 2016), reflecting differences among ecological communities in ecosystems with distinct characteristics (Andersen et al. 2015). These characteristics represent the ecological constraints of each location on the regional pool of species, exemplified by the quantity and variety of resources, conditions, and interactions among species (Loreau 2000).

Ants, owing to their high diversity, wide distribution, and rapid, predictable response to environmental and ecological changes, have been utilized as biological models in studies of ecology, macroecology, biogeography, and conservation biology (Wilson and Holldobler 2005; Lach and Kirsti 2010; Griffiths et al. 2018). Thus, the diversity of ant species on a global scale is determined by climate and landscape characteristics (Arnan et al. 2015; Andersen et al. 2015; Brasil et al. 2018; de Castro et al. 2020; Dantas and Fonseca 2023). In some ecosystems within the Neotropics, drivers such as altitude, precipitation, and temperature have been identified as drivers of ant species diversity at regional scales (Silva and Brandão 2010; Vasconcelos et al. 2018; Lasmar, Bishop, et al. 2021).

Ant diversification in the Neotropical region is often linked to forest habitats (Moreau and Bell 2013). Thus, numerous species inhabit forest ecosystems (BAH! 2023), and several species can live in both forest and open ecosystems, which characterizes them as generalists (Vasconcelos et al. 2018), while a few species are specialists in open ecosystems (Leal et al. 2017). These habitat‐use guilds offer a clearer perspective on how environmental changes, whether natural (Vasconcelos et al. 2018) or anthropogenic (Martins et al. 2022; Dutra et al. 2023), affect ant assemblage composition. Forest specialist ants tend to increase in species richness with precipitation (Vasconcelos et al. 2018), but their richness decreases in areas with low forest cover (Martins et al. 2022; Dutra et al. 2023). On the other hand, open habitat specialists and generalists tend to increase species richness in environments with low precipitation (Vasconcelos et al. 2018) and at high levels of disturbance (Paolucci et al. 2017; Martins et al. 2022).

The Brazilian Amazon, the largest continuous area of tropical rainforest in the world, exhibits significant climatic and vegetation variation (Artaxo 2023). It is also a landscape in constant transformation, marked by a prominent deforestation gradient (INPE 2023), which has important implications for global climate changes (Monteverde et al. 2022; Papastefanou et al. 2022). These changes are driven by land‐use conversion systems and the abandonment of areas previously dedicated to agriculture and pastures (Nagendra et al. 2004; Carvalho et al. 2017; Chaddad et al. 2022). Its southwestern portion, specifically in the state of Acre, stands out as a notable example of a region with remarkable contrasts in both precipitation and forest cover (Acre 2010). This variation is notable, ranging from the east, where the region is drier, to the west, where it becomes more humid (Duarte 2006). The same applies to the proportion of forest cover, with the eastern experiencing a more pronounced deforestation level compared to the western (Acre 2010; INPE 2023). Additionally, the southwestern Brazilian Amazon has been pointed out as a shortfall in biodiversity knowledge (Carvalho et al. 2023).

In this context, our study represents the first documented effort in southwestern Brazilian Amazon (Schmidt et al. 2020) to investigate the drivers of ant diversity at a regional scale, considering not only climatic drivers but also the impact of forest cover on ant diversity patterns. Our objective was to examine the influence of precipitation and forest cover proportion on ant species richness and composition. We expect a positive effect of forest cover and precipitation on ant species richness (Vasconcelos et al. 2018; da Costa and Schmidt 2022; Parr and Bishop 2022).

Additionally, we will examine this effect for habitat‐use guilds, where forest specialist richness should respond positively to increased precipitation and forest cover, open habitat specialists negatively, and generalists should not exhibit a significant response (Vasconcelos et al. 2018; Martins et al. 2022; Dutra et al. 2023). For ant species composition, we expect to observe differences among the sampled ant assemblages, driven by variations in precipitation levels and forest cover proportions (da Costa and Schmidt 2022). However, differences in species composition between two assemblages may also be influenced solely by the spatial distance between them (Soininen et al. 2018; Martins et al. 2022). Thus, we determined whether the change in species composition among ant assemblages is due to climatic and forest cover differences or simply by the spatial distance among them. Finally, to better understand the mechanisms underlying these compositional shifts, we evaluated beta diversity by decomposing it into turnover and nestedness components. We hypothesize that turnover will be the dominant process driving differences in species composition (Soininen et al. 2018), as environmental filtering should favor species replacements along gradients of precipitation and forest cover.

## 2 Materials and Methods

### 2.1 Study Site

We conducted our study in the State of Acre, located in the southwestern Brazilian Amazon. Acre exhibits considerable climatic and environmental variation that is oriented by a geographical eastern‐western gradient. Thus, there is a notable variation regarding precipitation and forest cover conservation (Acre 2010) where the eastern region is drier than the western (Duarte 2006). Additionally, the eastern region experiences a more pronounced deforestation level compared to the western (Acre 2010; INPE 2023).

The vegetation types are also organized along this geographical gradient, providing the state with a wide diversity of forest types (Daly and Silveira 2008; Daly et al. 2016). This diversity ranges from upland forests with the presence of bamboo and palm trees, dense forests with continuous canopy, to vegetation complexes on white sand, known as ‘campinas’ and ‘campinaranas’ (Acre 2010; Daly et al. 2016). The state has an equatorial climate, hot and humid, with an average annual precipitation of 2200–2500 mm, with January being the rainiest month and July the driest (Alvares et al. 2013). The soils of the state are predominantly clay‐sandy sediments from the Solimões formation, covering almost the entire territory (80%), except for the northwest region where Paleozoic and Mesozoic rocks predominate (Bardales et al. 2010; Acre 2010). The relief of Acre is predominantly flat, with little altitude variation throughout the state, ranging from about 300 m at the international borders to just over 110 m at the boundaries with the State of Amazonas (Acre 2010). The hydrography of the state is characterized by the presence of four major basins—Acre, Purus, Juruá, and Tarauacá—flowing towards the Amazon (Acre 2010), which were used to guide the collection logistics throughout the state.

### 2.2 Sampling Design

**FIGURE 1:**
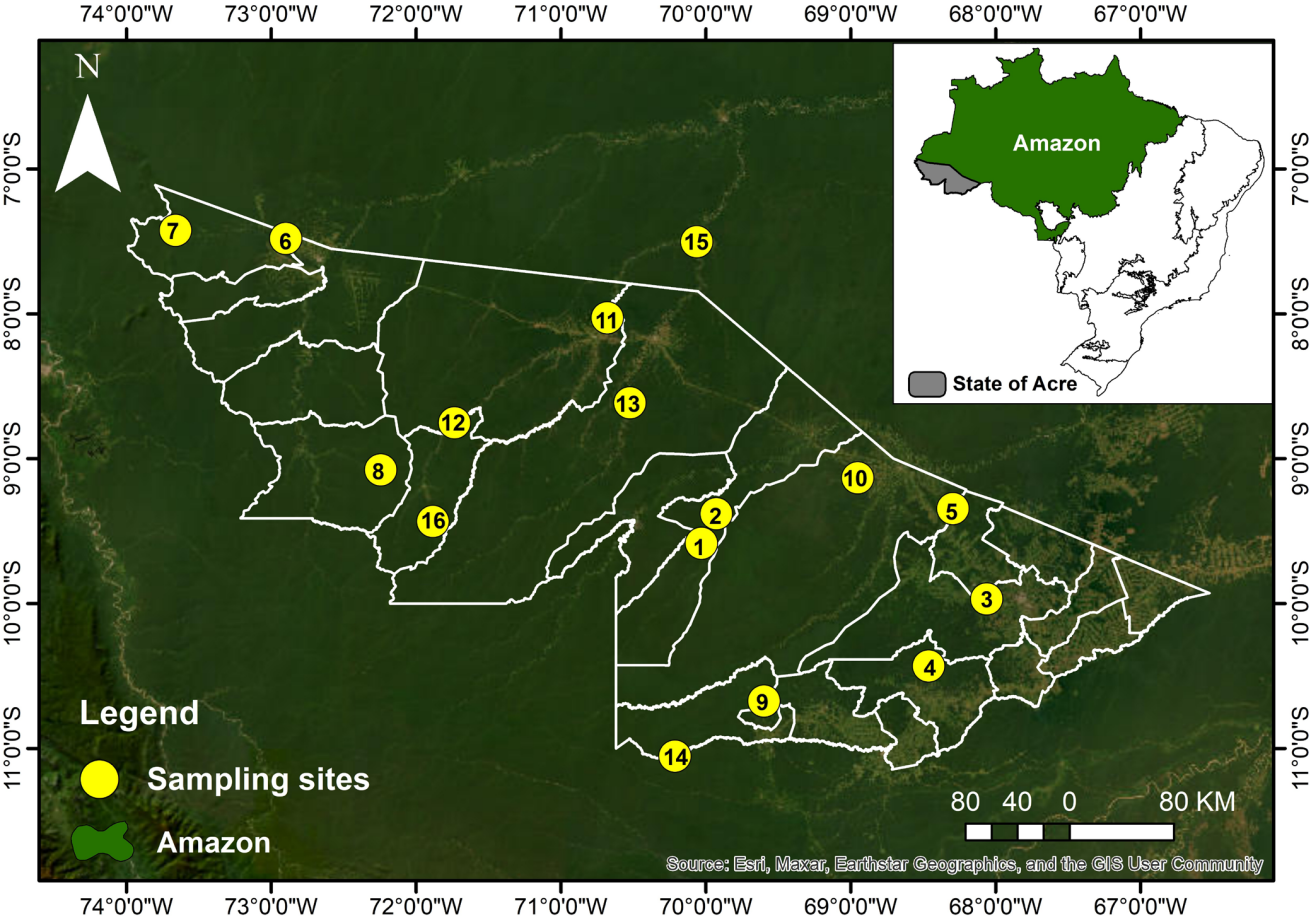
Geographical distribution of the 16 sampling areas throughout the State of Acre—Southwestern Brazilian Amazon. The samplings were carried out from 2014 to 2019. The area numbers correspond to those listed in Table S1.

The sampling areas (*n* = 16) (Table S1) were delineated from east to west in the state of Acre to achieve a geographically representative range (Figure 1) and capture the respective bioclimatic variations and forest cover changes in the study region (see more above), including very remote areas.

Ant samplings were carried out from 2014 to 2019, occurring in the dry season (between May and August) and in the rainy season (between December and March). According to Queiroz et al. (2023) for the Amazon biome there are no effects of seasonality on ant diversity. Thus, this logistical approach was necessary because some areas are accessible only by land, which becomes nearly impossible during the rainy season. Conversely, other areas are accessible only by rivers, and during the dry season, the river levels become too low, making access challenging.

### 2.3 Ant Sampling and Identification

The sampling areas were always in forest habitat with a high level of canopy cover (previously checked in Google Earth). Additionally, with the help of local residents the location was chosen for the sampling area where they reported it to be a primary forest without any intensive human use (e.g., woody logging and animal hunting). The transects were established starting 100 m from the edge of the forest. Along each transect, 10 plots of 25 m^2^ (5 m × 5 m) were set up. The centers of these plots were spaced 20 m apart, resulting in a total transect length of approximately 230 m.

In each plot, we installed one pitfall trap on the ground surface, one on tree trunks, and one underground (Bestelmeyer and Wiens 2001; Ribas et al. 2003; Schmidt and Solar 2010) and collected a 1 m^2^ litter sample (Bestelmeyer and Wiens 2001). The pitfall traps consisted of 300 mL plastic containers containing a lethal solution composed of water, glycerol (5%), and salt (0.9%), which remained in the field for 48 h. Ants present in the litter sample were collected using a mini‐Winkler extractor, involving a 48‐h fauna extraction period (Bestelmeyer and Wiens 2001). The ants collected in both the pitfall traps and the litter together formed the ant sample for each plot, while the set of ants collected in the ten plots formed the ant assemblage for each sampling area, which resulted in 16 sample units.

All sampled ants were stored in containers with 90% alcohol for sorting, mounting, and identification in the laboratory. Specimens of each morphospecies from each sample were separated for mounting, forming a physical collection and project voucher specimens.

We used taxonomic keys available in Baccaro et al. (2015) for ant genera identification. Ants belonging to the same genus were separated into morphospecies based on the similarity of external morphology. Species‐level identification was conducted at the Museum of Zoology, University of São Paulo—MZUSP, and through comparisons with specimens from the Ant Collection of the Insect Ecology Laboratory, Federal University of Acre—UFAC. Species‐level determinations were carried out by ant taxonomists (see Acknowledgement). All collected ants were deposited in the Ant Collection of the Insect Ecology Laboratory at UFAC.

### 2.4 Set of Explanatory Variables

To obtain precipitation data, we selected the following bioclimatic variables: annual precipitation, precipitation of the wettest month, and precipitation of the driest month, considering that precipitation is reported as an influencing factor on ant richness (Vasconcelos et al. 2018; Parr and Bishop 2022) (Table S1). We manually extracted values of bioclimatic variables from each raster using the “*extract*” function from the R package “*raster*” (Hijmans 2020). This process involved obtaining values using the geographical coordinates of the sampling areas as reference points. The bioclimatic variables consist of sets of raster layers with a spatial resolution of 1 km^2^, available in WorldClim (Fick and Hijmans 2017).

To assess the forest cover surrounding the sampling areas, we measured its proportion based on land use and land cover maps provided by MapBiomas (2023), using the “*multi.land.mtrc*” function in the R package “*spatialEco*” (Evans 2021). The analysis was based on the MapBiomas Vegetation Degradation Module for Native Vegetation in Brazil (1986–2021, beta version), which provides data at a 30‐m spatial resolution.

To do this, we created a buffer with a radius of 500 m from the central point of the transect. We considered the forest habitat within each circular area as the amount of forest vegetation present. This radius was chosen as it represents a biologically relevant geographic area for ants to interact with landscape elements through alate dispersal (Hölldobler and Wilson 1990) and also for the coexistence of ant species in the area and their ability to disperse between different habitat types (Spiesman and Cumming 2008; Schmidt et al. 2017). The extraction of the described variables (annual precipitation, precipitation of the wettest month, and precipitation of the driest month and forest cover) was performed in the R program version 4.3.0 (R Core Team 2023).

### 2.5 Habitat‐Use Guilds Definition

To classify the ant fauna according to habitat‐use guilds, we only considered ants identified at the species level. This decision was made because ants identified at the species level provide more precise information about their habitat preferences, allowing for a more reliable classification. Initially, we assessed habitat‐use guilds provided in BAH (2023), which is an application program that informs ecological information about Brazilian ant species, such as habitat‐use guilds. For ant species whose habitat‐use guild was not available, we applied the method proposed by Dutra et al. (2023). We used the habitat types listed under the “Habitat summary” section on AntWeb.org, applying an 80% threshold: a species was classified as a forest or open‐habitat specialist when occurrences in one of these habitat types accounted for 80% or more of the records. Species were considered generalists when their occurrences in any specific habitat type were below this threshold (Dutra et al. 2023).

### 2.6 Data Analysis

All analyses were performed in the R program version 4.3.3 (R Core Team 2023). Initially, we tested the normality of the data using the Shapiro–Wilk normality test. Subsequently, we performed Spearman correlation tests among the three bioclimatic variables: annual precipitation (Bio 12), precipitation of the wettest month (Bio 13), and precipitation of the driest month (Bio 14). We established a reference criterion of 0.7 (*p* < 0.05) for correlation, meaning that if the correlation between variables exceeded this value, we opted to select the variable we considered biologically more capable of explaining the observed patterns and having its use reported in previous studies (Vasconcelos et al. 2018).

We found a high positive correlation between annual precipitation (Bio 12) and precipitation of the wettest month (Bio 13) (0.95). Therefore, we decided to use only annual precipitation, as it was also reported as an explanatory variable with significant influence in previous studies on ant diversity at a regional scale (Vasconcelos et al. 2018; Lasmar, Bishop, et al. 2021). Additionally, we considered precipitation of the driest month as an explanatory variable in the models, as it showed a low correlation with annual precipitation (0.65). The correlations between the selected variables are summarized in Table S2.

To assess the influence of annual precipitation, precipitation of the driest month, and forest cover on total ant species richness, we constructed Generalized Linear Models (GLMs). We used the total ant species richness found in each sampling area as the response variable and annual precipitation, precipitation of the driest month, and forest cover proportion as explanatory variables. This model had a *Poisson* error distribution, and due to overdispersion observed in the residual analysis, we used a negative Binomial error distribution (Crawley 2013).

Subsequently, to examine the effect of annual precipitation, precipitation of the driest month, and forest cover on species richness of ant habitat‐use guilds, we employed a Generalized Linear Model (GLM), where species richness was the response variable and the same variables described above were the explanatory variables. Furthermore, the interaction between habitat‐use guilds and the three explanatory variables was considered in the model. The model had a Poisson error distribution (Crawley 2013). We utilized the *backward selection regression* approach for model simplification. Finally, if any explanatory variable showed a significant influence on total species richness or on species richness of habitat‐use guilds, we tested which model better explained this relationship, the linear model or the “*broken stick*” model with threshold, using the function from Nagai (2011). We used Akaike's Information Criterion (AIC) as the selection criterion, obtained through the R package “*AICcmodavg* 2.3‐4” (Mazerolle 2023). If the difference between the AICs is greater than two, we choose the model with the lower AIC. If the difference is less than two, both models explain equally; thus we opted for the simpler model.

To estimate the differences on species composition of ants among the sampling areas, we calculated β diversity using the Sørensen dissimilarity index, obtained through the R package “*betapart* 1.6” (Baselga and Orme 2012) and utilizing the *“beta.pair”* function. The Sørensen dissimilarity index is a measure that ranges from identical sets (0) to completely different sets (1). We also partitioned βsør into its components: turnover (βsim) and nestedness (βnes), through the R package “*betapart* 1.6”, where βsim and βnes range from 0 (0%) to 1 (100%), indicating their relative contribution to βsør. βsim refers to species replacement, and βnes refers to species gain or loss (Baselga 2010).

We used partial Mantel tests to evaluate whether differences in forest cover, annual precipitation, precipitation of the driest month, and spatial distance between sampling areas influence βsør diversity and its components βsim and βnes (Legendre and Legendre 2012).

We individually estimated differences in annual precipitation, precipitation of the driest month, and forest cover with the Euclidean distance of these variables between pairs of sampling locations, generating three matrices, one for each analyzed variable. We estimated spatial distance as the Euclidean distance between pairs of sampling locations using longitude and latitude in decimal degrees.

We ran a partial Mantel test to estimate the effect of a predictor matrix (differences in annual precipitation, precipitation of the driest month, forest cover, or spatial distance) on a response matrix (βsør dissimilarities, βsim, or βnes), while controlling for the effect of a third matrix (differences in annual precipitation, precipitation of the driest month, spatial dissimilarity, or differences in forest cover) (Legendre and Legendre 2012). We estimated the partial Mantel association with Pearson's coefficient and assessed its significance with 9999 permutations. We calculated partial Mantel tests separately for each response matrix for βsør, βsim, or βnes dissimilarities. All analyses were conducted in R using the packages “*vegan*” 2.6.6 (Oksanen et al. 2025), “*betapart*” 1.6 (Baselga et al. 2025), “*ggplot2*” 3.5.2 (Wickham 2016), and “*ggpubr*” 0.6.0 (Kassambara 2025).

We used variation partitioning to assess the contribution of differences in annual precipitation, precipitation of the driest month, forest cover, and spatial distances between sampling areas to calculate the proportion of variance of the dissimilarity indices of multiple sites. To do this, we performed variation partitioning with distance‐based redundancy analysis (dbRDA; Legendre and Anderson 1999) using dissimilarities of multiple sites as the response matrix and differences in annual precipitation, precipitation of the driest month, forest cover, and longitude of the sampling location as explanatory matrices. Like the partial Mantel described above, we estimated the individual effect of each explanatory matrix on the response matrix, controlling for the effects of the other explanatory matrices. We described spatial distance only with longitude to avoid collinearity between latitude and longitude in dbRDA. We assessed the significance of individual fractions with 9999 permutations. The dbRDA was performed using the R package “*vegan*” (version 2.6‐6; Oksanen et al. 2025).

We assessed sampling completeness using species accumulation curves for each sampling area (*n* = 10), plotting the estimated number of species (Chao2) against the number of samples (see Figure S1). We performed 999 randomizations with replacement to generate confidence intervals around the accumulation curves. Because ant assemblages contain many rare species (i.e., species recorded in only one sample), we used the Chao2 estimator, which accounts for rare species and can be applied to presence–absence matrices (Magurran 2011).

## 3 Results

### 3.1 Ant Fauna and Habitat‐Use Guilds

We collected 365 ant species, with 41% identified at the species level and 59% sorted into morphospecies. The ant species belonged to 59 genera and are distributed across eight subfamilies, with Myrmicinae being the subfamily with the highest number of species (215 species), followed by Formicinae (50), Ponerinae (45), Dolichoderinae (21), Ectatomminae (17), Dorylinae (7), Pseudomyrmecinae (6), Amblyoponinae (2), Proceratiinae (2), and Paraponerinae (1). Among the collected species, *Pseudomyrmex crudelis*, *Strumigenys longispinosa* , *Strumigenys gundlachi* and *Stegomyrmex connectens* , were recorded for the first time in Brazil based on the samplings conducted in this study (Janicki et al. 2016). Additionally, we recorded 33 new records of ant species in the State of Acre (Table S3).

However, two species could not be classified due to a lack of available information: *Apterostigma jubatum* Wheeler, 1925, and *Paratrachymyrmex diversus* Mann, 1916.

### 3.2 Ant Species Richness

**FIGURE 2:**
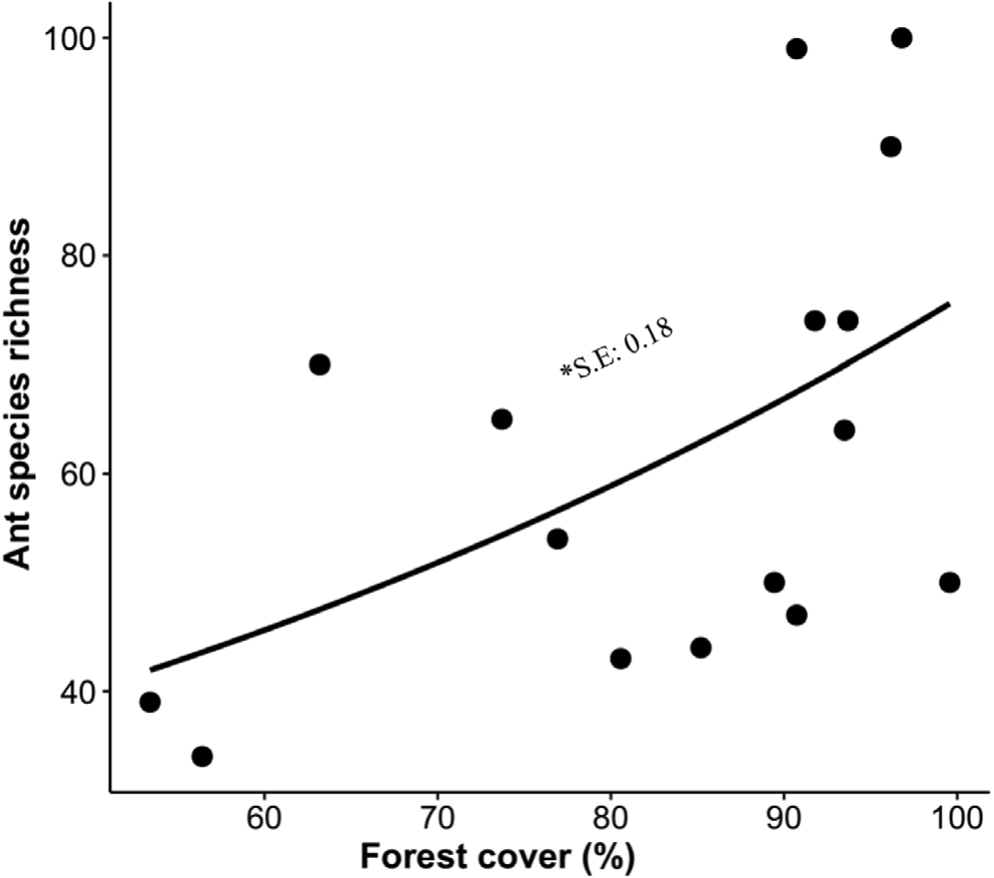
Relationship between total ant species richness and forest cover proportion (*ꭓ* ^2^_(1,14)_ = 6.63; *p* < 0.01; *R* ^2^ = 0.294). Acre, southwestern Brazilian Amazon. *S.E, standardized estimate.

The total species richness of ants varied from 39 to 100 species per sampling area (mean ± SD 62.31 ± 20.87 species). Among the set of explanatory variables (annual precipitation, precipitation of the driest month, and forest cover), we found only a positive relationship between total ant species richness and percentage of forest cover (*ꭓ* ^2^_(1,14)_ = 6.63; *p* < 0.01; Figure 2), thus the non‐significant explanatory variables were removed from the final model.

Considering the 151 species classified according to their habitat‐use guilds, ant species richness of forest specialists ranged from 8 to 32 (mean ± SD 19.94 ± 6.32 species), while generalist species richness ranged from 4 to 16 (mean ± SD 10.12 ± 3.18 species), and open‐area specialist species ranged from 0 to 2 (mean ± SD 0.62 ± 0.62 species).

**FIGURE 3:**
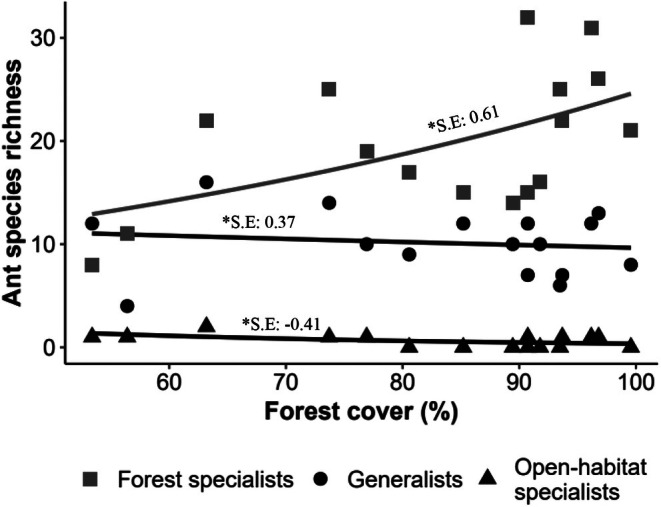
Species richness of three habitat‐use ant guilds with forest cover proportion in southwestern Brazilian Amazon. Effects on ant species (*Ϝ*_(2,42)_ = 8.92; *p* < 0.01). *S.E, standardized estimate. Square: Forest specialists, Circle: Generalists and Triangle: Open‐area specialists.

Regarding the model constructed to verify the effect of annual precipitation, precipitation of the driest month, and forest cover on species richness of ant habitat‐use guilds, only forest cover (*F*_(1,46)_ = 4.31; *p* < 0.03), habitat‐use guilds (*F*_(2,44)_ = 366.59; *p* < 0.05) and the interaction (*Ϝ*_(2,42)_ = 8.92; *p* < 0.01) between these two variables presented significant effects on species richness. Thus, forest ant specialists increased the species richness with forest cover, while open‐area ants and generalists present a low variation with forest cover (Figure 3). We selected the model with the lowest AIC to describe the relationship between forest cover and species richness of habitat‐use guilds (Figure S2). For forest specialists and open‐area specialists, the linear model had a lower AIC than the “*broken stick*” model (Figures S3 and S4). For generalist species, the AIC difference between models was less than two, indicating similar explanatory power; thus, we opted for the linear model (Figure S5).

### 3.3 Ant Species Composition

**FIGURE 4:**
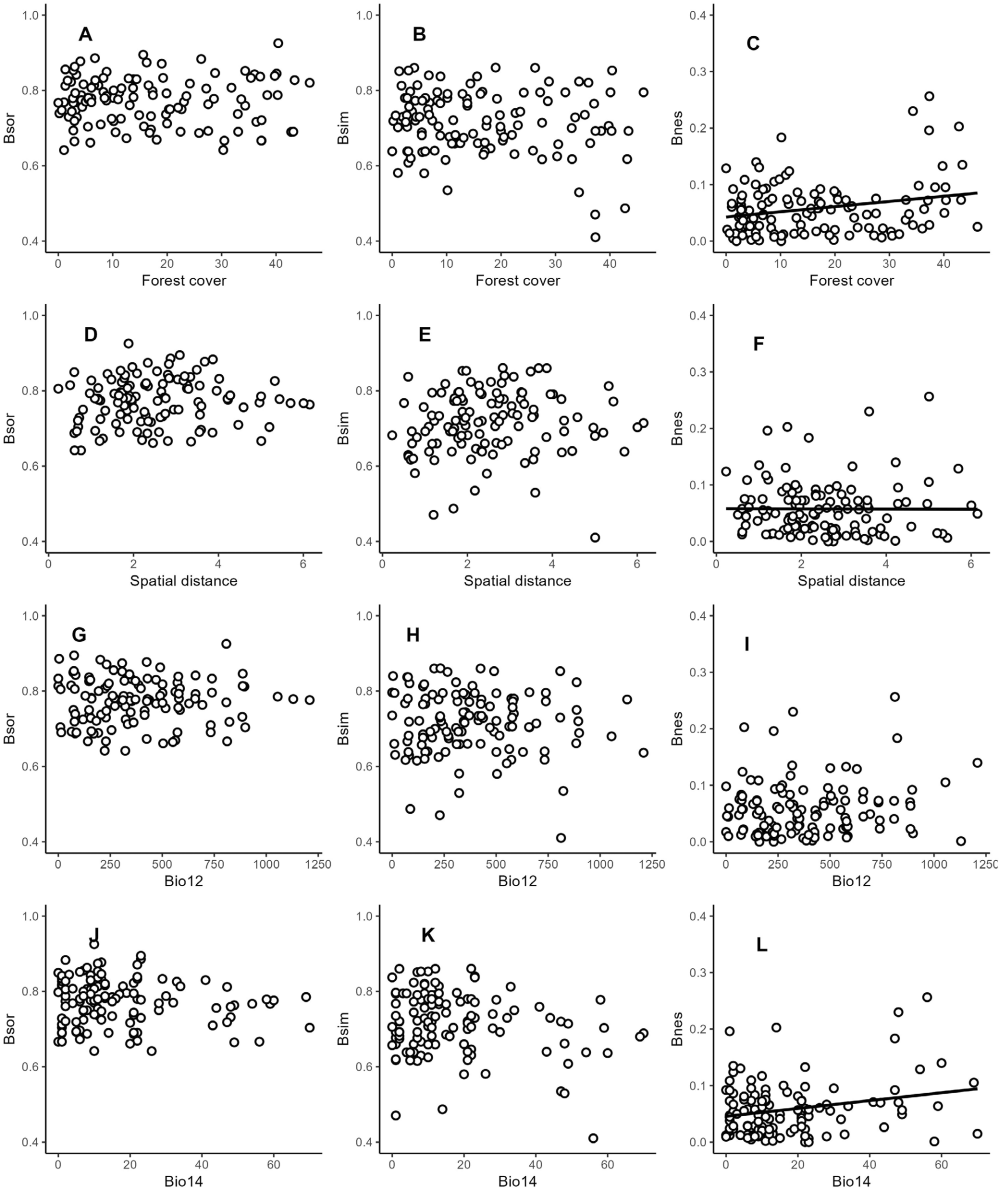
Relationship between forest cover difference (A–C), spatial distance (D–F), annual precipitation (Bio 12) (G–I), or precipitation of the driest month (Bio 14) (J–L). Each row shows the βsør dissimilarity and its components, βsim, and βnes. The lines represent trend lines for statistically significant relationships.

The βsør diversity ranged from 0.64 to 0.92 (mean ± SD 0.77 ± 0.06), while its turnover component (βsim) ranged from 0.41 to 0.86 (mean ± SD 0.71 ± 0.08), and its nestedness component (βnes) ranged from zero to 0.25 (mean ± SD 0.04 ± 0.04), suggesting that a large portion of dissimilarity was due to species turnover among the ant assemblages. We found a positive correlation between βnes and forest cover (Figure 4C) and with precipitation in the driest month (Figure 4L) when controlling for spatial distance (Table 1). This means that the difference in species composition between ant assemblages due to differences in forest cover and rainfall in the dry season among sampled areas occurs due to the nestedness component. We also found that the greater the spatial distance between sampling areas, the higher the βsim (Figure 4F) while controlling for differences in precipitation in the driest month (Table 1). This means that as the distance between locations increases, species communities become more distinct from each other, driven by the species turnover component even when conditions of rainfall in the driest month are similar.

**TABLE 1:** Partial Mantel test evaluating the relationship between forest cover difference and βsør, βsim, and βnes dissimilarities across multiple sites while controlling for spatial distance [r M (ForCov | Spa)] (Figure 4A–C) and between spatial distance and βsør, βsim, and βnes dissimilarities across multiple sites while controlling for forest cover difference [r M (Spa | ForCov)] (Figure 4D–F). We also assessed the relationship between annual precipitation difference and difference in precipitation in the driest month among sites and βsør, βsim, and βnes dissimilarities across multiple sites while controlling for spatial distance [r M (Bio12 | Spa); r M (Bio14 | Spa)] (Figure 4G–L) and between spatial distance and βsør, βsim, and βnes dissimilarities across multiple sites while controlling for the difference in annual precipitation (Bio 12) and precipitation in the driest month (Bio 14). Statistically significant partial Mantel correlations (r M) are in bold. ForCov: Forest Cover and Spa: Spatial Distance.

Response matrix	Forest cover difference	*p*	Difference in Bio 12	*p*	Difference in Bio 14	*p*
	*r M* (ForCov | Spa)		*r M* (Bio12 | Spa)		*r M* (Bio14 | Spa)	
Βsør	−0.01869	0.5588	−0.000165	0.4969	−0.2016	0.9038
Βsim	−0.1542	0.8947	−0.07995	0.7508	−0.3211	0.9915
βnes	0.2462	**0.0294**	0.1396	0.1104	0.3058	**0.0125**

	*r M* (Spa | ForCov)		*r M* (Spa| Bio12)		*r M* (Spa| Bio14)	
Βsør	0.04755	0.3502	0.0463	0.3347	0.17	0.06
Βsim	0.02306	0.4319	0.05074	0.3251	0.2386	**0.0094**
βnes	0.02118	0.3871	−0.03005	0.5868	−0.1982	0.9922

The spatial distance explained a larger proportion of the variance in βsør diversity and its turnover component (βsim) than forest cover (Table 2). We also observed that spatial distance, when analyzed controlling for annual precipitation (Bio12), also explained a greater proportion of the variance for βsør diversity (Table 2). This suggests that the geographical location of the studied areas played a more significant role in explaining changes in ant species composition than forest cover itself and precipitation. However, for the nestedness component (βnes), differences in forest cover, annual precipitation, precipitation of the driest month, and spatial distance did not explain the variance proportion.

**TABLE 2:** Variation partitioning assessing the individual and shared contribution of forest cover difference and βsør diversity and its components βsim and βnes while controlling for spatial distance (ForCov | Lon) and between spatial distance and βsør diversity and its components βsim andβnes while controlling for forest cover difference (Lon | ForCov). Individual and shared contribution of annual precipitation difference (Bio12) and βsør diversity and its components βsim and βnes while controlling for spatial distance (Lon | Bio12) and between spatial distance and βsør diversity and its components βsim and βnes while controlling for annual precipitation difference (Bio12 | Lon). Individual and shared contribution of the difference in the precipitation of the driest month (Bio14) and βsør diversity and its components βsim and βnes while controlling for spatial distance (Lon | Bio14) and between spatial distance and βsør diversity and its components βsim and βnes while controlling for the difference in precipitation of the driest month (Bio14 | Lon). Statistically significant adjusted coefficients of determination (*R* ^2^ adj) are in bold. ForCov: Forest cover and Lon: Spatial distance.

Response matrix	Explanatory matrix	*R* ^2^ adj	*p*
Bsor (total)	ForCov | Lon	0.05728	0.648
Bsor (total)	Lon | ForCov	0.08888	**0.038**
Bsor (total)	Shared fraction	0.14880	
Bsim (turnover)	ForCov | Lon	0.03702	0.878
Bsim (turnover)	Lon | ForCov	0.10261	**0.02**
Bsim (turnover)	Shared fraction	0.14717	
Bsne (nestedness)	ForCov | Lon	0.41119	0.08
Bsne (nestedness)	Lon | ForCov	0.04229	0.621
Bsne (nestedness)	Shared fraction	0.34008	
Bsor (total)	bio12 | Lon	0.07142	0.354
Bsor (total)	Lon | bio12	0.08888	0.065
Bsor (total)	Shared fraction	0.15834	
Bsim (turnover)	bio12 | Lon	0.07847	0.225
Bsim (turnover)	Lon | bio12	0.10261	**0.032**
Bsim (turnover)	Shared fraction	0.17982	
Bsne (nestedness)	bio12 | Lon	−0.04945	0.062
Bsne (nestedness)	Lon | bio12	0.04229	0.645
Bsne (nestedness)	Shared fraction	−0.03210	
Bsor (total)	bio14 | Lon	0.06785	0.848
Bsor (total)	Lon | bio14	0.08888	0.279
Bsor (total)	Shared fraction	0.14157	
Bsim (turnover)	bio12 | Lon	0.06699	0.804
Bsim (turnover)	Lon | bio14	0.10261	0.132
Bsim (turnover)	Shared fraction	0.15183	
Bsne (nestedness)	bio12 | Lon	0.13986	0.479
Bsne (nestedness)	Lon | bio14	0.04229	0.649
Bsne (nestedness)	Shared fraction	0.09443	

## 4 Discussion

In this study, increased forest cover was associated with greater total species richness and more forest‐specialist ants. This highlights the sensitivity of ant species richness to forest habitat amount (Ahuatzin et al. 2019; da Costa and Schmidt 2022) but the habitat‐use guilds respond distinctly (Martins et al. 2022). Spatial distance plays a significant role in explaining the variance in βsør diversity and its turnover component (βsim), surpassing the contribution of forest cover. However, for the nestedness component (βnes), even when controlling for the influence of geographic distance, variability in forest cover and precipitation of the driest month still exerts a direct influence on ant species composition among the ant assemblages. Thus, at a regional scale, the forest cover conservation level surrounding the ant assemblage sampled is a more significant driving factor than precipitation‐related variables for species richness. In turn, species composition variation among ant assemblages occurs due to increased geographic distance rather than differences in forest cover and precipitation.

### 4.1 Ant Fauna

The collected data reveal the great diversity of ants in the southwestern Brazilian Amazon. Considering the number of ant species sampled (365), this study stands out as the most comprehensive survey ever conducted on ant diversity in the region (Schmidt et al. 2020). Furthermore, it is worth noting that this study was pioneering in sampling ants across the entire territory of Acre, significantly contributing to the advancement of knowledge about ant diversity and distribution (Carvalho et al. 2023). Our sampling effort also resulted in 33 new ant species records for the state of Acre and four for Brazil, reinforcing the importance of biodiversity surveys in under‐sampled regions (Schmidt et al. 2020; Carvalho et al. 2023; Dutra et al. 2023).

### 4.2 Ant Species Richness, Habitat‐Use Guilds, and Environmental Variables

Among the variables analyzed (forest cover, annual precipitation, and precipitation of the driest month) in this study, we found a positive relationship between changes in forest cover and ant species richness. Forest cover proportion has been identified as one of the main drivers of ant species diversity at large scale (Solar et al. 2016; Ahuatzin et al. 2019; da Costa and Schmidt 2022; Martins et al. 2022). This observed positive relationship can be attributed to the greater environmental heterogeneity found in landscapes with higher forest cover, which leads to an increase in habitat variation, resource availability, and breeding sites (Ribas et al. 2003; Stein et al. 2014; Ortega et al. 2018). In contrast, ant richness tends to decline in landscapes where drastic changes in vegetation structure have occurred, particularly in land‐use conversions involving strong contrasts, such as the replacement of tropical forests with anthropogenic areas (Dantas and Fonseca 2023; Larrea et al. 2024; Wilker et al. 2024). However, it is worth noting that this species richness of ants is affected differently depending on the habitat‐use guild. We observed that forest cover positively affects only forest specialists and not open‐area specialists and generalists. This can be explained by the fact that the majority of ant species in the Neotropical region have their diversification associated with forest habitat (Moreau and Bell 2013). In other words, the three‐dimensional structure of forests allows for vertical stratification, promoting niche differentiation and facilitating species coexistence (Dejean et al. 2024). Furthermore, leaf litter plays a fundamental role in shaping ant communities. Areas with deeper and more heterogeneous litter layers tend to support more individuals and species, as they offer more nesting sites, prey availability, and microclimatic protection (Moses et al. 2021). For example, forests exhibit the highest densities of leaf‐litter ants, whereas arid environments harbor a greater number of ants that actively forage on the ground (Schultheiss et al. 2022). These patterns reflect how specific ant characteristics—such as nesting in soil, leaf litter, or decaying wood, and foraging in moist and shaded microhabitats—are closely linked to forest structure and cover.

As for the ants identified as generalists, we can observe that they are present in all sampling areas, showing no significant variations between these areas, indicating that they are less affected by changes in forest cover. This characteristic can be attributed to the ability of some generalist species to tolerate a wide range of environmental conditions, enabling them to exhibit plasticity that allows them to inhabit both forested and open habitats (Olden and Rooney 2006; Vasconcelos et al. 2018; Kramer et al. 2023). This adaptive capacity may give these species an advantage in the face of changes in land use and land cover (Paolucci et al. 2017; Martins et al. 2022; Dutra et al. 2023). Finally, the richness of open area specialists remained low across all sampling areas; however, this habitat‐use guild may surpass forest specialist richness in landscapes with low forest cover (e.g., below 20%) (Martins et al. 2022), in forest areas degraded by fire (Paolucci et al. 2017), and pasture areas (Sales and Schmidt 2023; Dutra et al. 2023).

### 4.3 Ant Species Composition and Environmental Variables

We observed that the variation in ant assemblage composition is more influenced by geographic location than by specific habitat characteristics, such as forest cover or variations in precipitation. Spatial distance played a significant role in explaining the variance in β‐diversity and its turnover component (βsim), surpassing the contribution of forest cover. This could be expected because we sampled the ants exclusively in forest habitat, that although for species richness, the proportion of around forest cover matters, the fact that the sampling areas present the same habitat type, forest cover was not a limiting factor for species composition.

The difference in species composition among ecological communities within a common landscape may arise from at least three processes: (i) differences in environmental characteristics, (ii) spatial configuration of the landscape, and (iii) limitations in the organisms' dispersal capacity (Soininen et al. 2018). Given that the variation in total beta diversity (βsør) and its turnover component (βsim) was primarily driven by spatial distance, we identify the limitation in the dispersal capacity of ant species (Hölldobler and Wilson 1990) as the primary mechanism for variation in species composition among the 16 ant assemblages sampled in the region. Of the 365 ant species collected, we noticed that 217 of them are restricted to one or two sample areas, indicating a significant limitation in the geographic dispersal capacity of these species (but the species accumulation curves presented a non‐saturation trend, which suggests that sampling limitation can be responsible for the restricted distribution of these species) Additionally, only four species demonstrated a broader distribution, occurring in more than 10 sample areas. These four species are *Neoponera apicalis*, *Mayaponera constricta*, and *Camponotus atriceps* , occurring in 11 areas, while the species *Odontomachus bauri* was found in 12 different areas.

In areas characterized by forested landscapes, where there is little variation in tree quantity and structure and canopy openness (i.e., little environmental variation) among the areas, the mechanism responsible for differences in species composition among assemblages may potentially be the limitation in the dispersal capacity of ant species (Schmidt et al. 2017). Soininen et al. (2007) reported that difference in species composition among natural communities could be due to processes of ecological drift, dispersal, and random speciation. The passage of four major hydrographic basins in the study region (Acre 2010) could be a factor that potentially contributes to restricting the dispersal capacity of ant species, making it as potential driver of differences on species composition among the ant assemblages, once the most part of ant species (i.e., 149 species) occurred in only one hydrographic basin.

## 5 Conclusion

Our findings provide valuable insights into the diversity and composition patterns of ants in the southwestern Brazilian Amazon. The association between increased forest cover and species richness, particularly for forest specialists, highlights the sensitivity of ant species to land use changes. However, geographic distance emerged as a primary factor in determining species composition variation, surpassing the influence of forest cover and precipitation variables at least in the spatial scale approached. Moreover, we can assert that the limitation in ant dispersal capacity reveals itself as a crucial mechanism in structuring the ant assemblages in the region. In this study, increased forest cover was associated with greater overall species richness and a higher number of forest‐specialist ants. The use of habitat‐use ant guilds favors a more detailed analysis and underscores the need for different approaches in management and conservation, considering the various response dynamics of ants to environmental factors, which can further enhance biodiversity preservation strategies in the studied region.

The richness and composition of natural communities are influenced by factors at both local and regional scales. While local forest cover stands out as a significant determinant of richness at the transect scale, regional analysis reveals the importance of dispersal and regional variables such as distance. Potential loss of forest cover at regional scales may intensify these dynamics, exacerbating impacts on species composition and overall biological diversity. This understanding not only broadens our insight into underlying ecological processes but also provides valuable insights for conservation and ecosystem management. In summary, by recognizing the interdependence between scales and processes, we are better equipped to develop effective conservation strategies that consider both local and regional aspects of biodiversity.

## Author Contributions

**Marília Maria Silva da Costa:** conceptualization (lead), data curation (lead), formal analysis (equal), investigation (equal), methodology (equal), writing – original draft (equal), writing – review and editing (equal). **Fernando Augusto Schmidt:** conceptualization (equal), formal analysis (equal), funding acquisition (lead), investigation (equal), methodology (lead), project administration (lead), resources (lead), writing – original draft (equal), writing – review and editing (equal). **Icaro Wilker:** data curation (equal), formal analysis (equal), visualization (equal), writing – original draft (equal), writing – review and editing (equal). **Chaim José Lasmar:** formal analysis (equal), investigation (equal), methodology (equal), writing – original draft (equal), writing – review and editing (equal). **Carla Rodrigues Ribas:** conceptualization (equal), formal analysis (equal), investigation (equal), methodology (equal), supervision (equal), writing – original draft (equal), writing – review and editing (equal).

## Conflicts of Interest

The authors declare no conflicts of interest.

## Supporting information


**Data S1:** ece372467‐sup‐0001‐Supinfo.zip.

## Data Availability

The data that support the findings of this study are openly available in Dryad Digital Repository: DOI: https://doi.org/10.5061/dryad.xksn02vt.
