# Supplementary material for: Forest Cover and Geographic Distance Shape Ant Assemblages in the Southwestern Brazilian Amazon
Source: Ecol Evol. 2025 Nov 12;15(11):e72467. doi: 10.1002/ece3.72467 (PMC12611350; doi:10.1002/ece3.72467)
Supplement: Supplementary file 1 — Data S1: ece372467‐sup‐0001‐Supinfo.zip. [file ECE3-15-e72467-s001.docx]

**FOREST COVER AND GEOGRAPHIC DISTANCE SHAPE ANT** **ASSEMBLAGES THE SOUTHWEST BRAZILIAN AMAZON**

Marília Maria Silva da Costa*, Fernando A. Schmidt ,Icaro Wilker, Chaim José Lasmar, Carla Rodrigues Ribas

**Corresponding author*:** [mariliamcostta@gmail.com](mailto:mariliamcostta@gmail.com)

**Table S1.** Location, forest formation, forest cover, and precipitation variables for areas where ant collections were conducted in the State of Acre, Brazil. The table includes information on municipality, watershed and its portion, sampling dates, forest formation type, geographic coordinates, percentage of forest cover, and bioclimatic precipitation variables: annual precipitation (Bio12), precipitation in the wettest month (Bio13), and in the driest month (Bio14).

| **Area** | **Municipality** | **Watershed** | **Watershed portion** | **Sampling dates** | **Forest formation*** | **Geographic coordinates** | **Forest cover (%)**** | **Bio12 (Annual Precipitation - mm)** | | **Bio13 (Rainy Month Precipitation - mm)** | **Bio14 (Dry Month Precipitation - mm)** |
| --- | --- | --- | --- | --- | --- | --- | --- | --- | --- | --- | --- |
| 1 | Manoel Urbano | Purus | High | 11/28/2014 | Open Alluvial Rainforest with Palms | 09º 35'02,4"S 70º01'55,0"W | 85,2 | 1376 | | 222 | 27 |
| 2 | Manoel Urbano | Purus | Medium | 12/01/2014 | Open Alluvial Rainforest with Palms | 09º 22'47,5"S 69º55'45,0"W | 96,8 | 1455 | | 236 | 29 |
| 3 | Bujari | Acre | Low | 11/18/2015 | Open Lowland Rainforest with Palms | 09º 58'00,7"S 68º03'47,5"W | 63,19 | 1892 | | 283 | 38 |
| 4 | Xapuri | Acre | Medium | 09/27/2016 | Open Lowland Rainforest with Bamboo | 10º 25'46,7"S 68º27'48,1"W | 93,49 | 2115 | | 339 | 28 |
| 5 | Sena Madureira | Purus | Low II | 04/15/2016 | Dense Lowland Rainforest with Bamboo | 09º 20'41,4"S 68º17'49,1"W | 90,73 | 1956 | | 273 | 33 |
| 6 | Cruzeiro do Sul | Juruá | Low | 06/07/2017 | Campinarana: Palm-free Forest | 07º 28'48,5"S 72º54'01,5"W | 91,78 | 2263 | | 290 | 61 |
| 7 | Mâncio Lima | Juruá | Medium | 09/19/2017 | Open Lowland Rainforest with Palms | 07º 25'27,1"S 73º39'42,4"W | 90,73 | 2585 | | 338 | 87 |
| 8 | Marechal Thaumaturgo | Juruá | High | 08/25/2017 | Open Lowland Rainforest with Bamboo | 09º 04'34,5"S 72º14'46,0"W | 80,57 | 1762 | | 268 | 40 |
| 9 | Assis Brasil | Acre | High | 06/15/2017 | Dense Lowland Rainforest | 10º 40'21,5"S 69º36'02,9"W | 96,17 | 1686 | | 240 | 17 |
| 10 | Sena Madureira | Purus | Low I | 07/15/2017 | Open Lowland Rainforest with Bamboo | 09º 07'53,0"S 68º57'12,6"W | 53,39 | 1773 | | 261 | 31 |
| 11 | Jordão | Tarauacá | Low | 05/03/2018 | Open Alluvial Rainforest with Palms | 08º 01'39,3"S 70º40'52,5"W | 76,92 | 2115 | | 321 | 46 |
| 12 | Jordão | Tarauacá | Medium | 05/28/2018 | Open Alluvial Rainforest with Palms | 08º 45'04,7"S 71º44'09,0"W | 89,44 | 1690 | | 250 | 40 |
| 13 | Feijó | Envira | High | 07/22/2018 | Open Lowland Rainforest with Palms | 08º36’48,1”S 70º31’28,9”W | 93,69 | 2034 | 320 | | 38 |
| 14 | Assis Brasil | Acre | High II | 04/08/2019 | Open Alluvial Rainforest with Palms | 11º03’06,6”S 70º12’55,6”W | 99,57 | 1530 | 220 | | 18 |
| 15 | Envira | Envira | Low | 05/18/2019 | Open Alluvial Rainforest with Palms | 07º30’07”S 70º03’54”W | 56,4 | 2263 | 340 | | 39 |
| 16 | Jordão | Tarauacá | High | 06/29/2019 | Open Alluvial Rainforest with Palms | 09º 25'58,4"S 71º53'8,53"W | 73,71 | 1700 | 260 | | 39 |

* Classification according to the Brazilian Institute of Geography and Statistics (IBGE), year 2016.

****Forest Cover (%)** was calculated as the proportion of forested area within a 500-meter radius circle centered on the transect midpoint. Land cover categories considered were those defined as native forest types in MapBiomas. The calculation was performed using the “*multi.land.mtrc*” function from the R package *spatialEco*, based on the MapBiomas Vegetation Degradation Module for Native Vegetation in Brazil (1986–2021, beta version) at 30-meter spatial resolution.

**Table S2.** Correlation between selected bioclimatic variables. Spearman and Pearson correlation coefficients (r or ρ) for the selected bioclimatic variables: annual precipitation (BIO12), precipitation of the wettest month (BIO13), and precipitation of the driest month (BIO14). Only correlations with a coefficient below 0.7 were considered.

| **Compared Variables** | **Method** | **Correlation Coefficient (r or ρ)** | ***p*-value** |
| --- | --- | --- | --- |
| Annual Precipitation (BIO12) ~ Precipitation of Wettest Month (BIO13) | Pearson | 0.906 | 1.35e-06 |
| Annual Precipitation (BIO12) ~ Precipitation of Driest Month (BIO14) | Spearman | 0.646 | 0.006891 |
| Precipitation of Wettest Month (BIO13) ~ Precipitation of Driest Month (BIO14) | Spearman | 0.553 | 0.0264 |

**Table S3.** List of ant species and habitat use guilds collected in the southwestern Brazilian Amazon. *First record of the species for the state of Acre (Janicki et al., 2023).

| **Species** | **Habitat Affinity** |
| --- | --- |
| **Amblyoponinae (2)** |  |
| Prionopelta aff. amabilis | - |
| Prionopelta aff. dubia | - |
| **Dolichoderinae (21)** |  |
| *Azteca* sp.1 | - |
| *Azteca* sp.2 | - |
| *Aztec*a sp.3 | - |
| *Azteca* sp.4 | - |
| *Azteca* sp.5 | - |
| *Dolichoderus attelaboides* (Fabricius, 1775) | Forest Specialist |
| *Dolichoderus bidens* (Linnaeus, 1758) | Forest Specialist |
| *Dolichoderus bispinosus* (Olivier, 1792) | Forest Specialist |
| *Dolichoderus decollatus* Smith, 1858 | Generalist |
| *Dolichoderus diversus* Emery, 1894 | Generalist |
| *Dolichoderus imitator* Emery, 1894 | Generalist |
| *Dolichoderus inermis* MacKay, 1993 | Forest Specialist |
| *Dolichoderus laminatus** (Mayr, 1870) | Generalist |
| *Dolichoderus lutosus* (Smith, 1858) | Generalist |
| *Dolichoderus mesonotalis* Forel, 1907 | Open Area Specialist |
| *Dolichoderus rugosus* (Smith, 1858) | Forest Specialist |
| *Dolichoderus septemspinosus* Emery, 1894 | Forest Specialist |
| *Dolichoderus* sp.1 | - |
| *Linepithema* sp.1 | - |
| *Linepithema* sp.2 | - |
| *Tapinoma melanocephalum* (Fabricius, 1793) | Generalist |
| **Dorylinae (7)** |  |
| *Eciton burchellii* (Westwood, 1842) | Generalist |
| *Eciton rapax* Smith, 1855 | Forest Specialist |
| *Labidus coecus* (Latreille, 1802) | Generalist |
| *Labidus praedator* (Smith, 1858) | Forest Specialist |
| *Labidus* sp.1 | - |
| *Neivamyrmex* sp.1 | - |
| *Nomamyrmex hartigii* (Westwood, 1842) | Generalist |
| **Ectatomminae (17)** |  |
| *Acanthoponera mucronata* (Roger, 1860) | Generalist |
| *Alfaria falcifera* (Kempf, 1967) | Forest Specialist |
| *Alfaria minuta* Emery, 1896 | Forest Specialist |
| *Ectatomma edentatum* Roger, 1863 | Generalist |
| *Ectatomma lugens* Emery, 1894 | Forest Specialist |
|  |  |
| **Continue*...*** |  |
| *Ectatomma tuberculatum* (Olivier, 1792) | Generalist |
| *Gnamptogenys acuminata* (Emery, 1896) | Generalist |
| *Gnamptogenys* cf. *continua* | - |
| *Gnamptogenys haenschi* (Emery, 1902) | Forest Specialist |
| *Gnamptogenys kempfi** Lenko, 1964 | Forest Specialist |
| *Gnamptogenys regularis* Mayr, 1870 | Forest Specialist |
| *Gnamptogenys* sp.1 | - |
| *Gnamptogenys* sp.2 | - |
| *Holcoponera moelleri* Forel, 1912 | Generalist |
| *Holcoponera relicta** (Mann, 1916) | Forest Specialist |
| *Holcoponera striatula* (Mayr, 1884) | Forest Specialist |
| *Poneracantha triangularis* (Mayr, 1887) | Forest Specialist |
| **Formicinae (50)** |  |
| *Acropyga* sp.1 | - |
| *Acropyga* sp.2 | - |
| *Brachymyrmex cavernicola* Wheeler, 1938 | Forest Specialist |
| *Brachymyrmex coactus** Mayr, 1887 | Forest Specialist |
| *Brachymyrmex* sp.1 | - |
| *Brachymyrmex* sp.2 | - |
| *Brachymyrmex* sp.3 | - |
| *Brachymyrmex* sp.4 | - |
| *Camponotus ager* (Smith, 1858) | Forest Specialist |
| *Camponotus atriceps* (Smith, 1858) | Generalist |
| *Camponotus bidens* Mayr, 1870 | Forest Specialist |
| *Camponotus blandus* (Smith, 1858) | Generalist |
| *Camponotus cacicus* Emery, 1903 | Forest Specialist |
| *Camponotus* cf. *bidens* | - |
| *Camponotus depressus* Mayr, 1866 | Forest Specialist |
| *Camponotus femoratus* (Fabricius, 1804) | Generalist |
| *Camponotus latangulus* Roger, 1863 | Forest Specialist |
| *Camponotus mirabilis* Emery, 1903 | Forest Specialist |
| *Camponotus rufipes* (Fabricius, 1775) | Generalist |
| *Camponotus* sp.1 | - |
| *Camponotus* sp.2 | - |
| *Camponotus* sp.3 | - |
| *Camponotus* sp.4 | - |
| *Camponotus* sp.6 | - |
| *Camponotus* sp.7 | - |
| *Camponotus* sp.8 | - |
| *Camponotus* sp.9 | - |
| *Camponotus* sp.10 | - |
| **Continue...** |  |
| *Camponotus* sp.12 | - |
| *Camponotus* sp.13 | - |
| *Camponotus* sp.14 | - |
| *Camponotus* sp.15 | - |
| *Camponotus* sp.16 | - |
| *Camponotus* sp.17 | - |
| *Camponotus* sp.18 | - |
| *Camponotus* sp.19 | - |
| *Camponotus* sp.20 | - |
| *Camponotus* sp.21 | - |
| *Camponotus* sp.22 | - |
| *Camponotus* sp.23 | - |
| *Camponotus* sp.24 | - |
| *Camponotus* sp.25 | - |
| *Camponotus* sp.26 | - |
| *Gigantiops destructor* (Fabricius, 1804) | Forest Specialist |
| *Nylanderia* sp.1 | - |
| *Nylanderia* sp.2 | - |
| *Nylanderia* sp.3 | - |
| *Nylanderia* sp.4 | - |
| *Nylanderia* sp.5 | - |
| *Nylanderia* sp.6 | - |
| **Myrmicinae (215)** |  |
| *Acromyrmex coronatus* (Fabricius, 1804) | Forest Specialist |
| *Acromyrmex hystrix** (Latreille, 1802) | Generalist |
| *Acromyrmex* sp.1 | - |
| *Acromyrmex* sp.2 | - |
| *Acromyrmex subterraneus* (Forel, 1893) | Open Area Specialist |
| *Apterostigma* aff. *peruvianum* | - |
| *Apterostigma auriculatum* Wheeler, 1925 | Forest Specialist |
| *Apterostigma ierense** Weber, 1937 | Forest Specialist |
| *Apterostigma jubatum* Wheeler, 1925 |  |
| *Apterostigma pilosum** Mayr, 1865 | Generalist |
| *Apterostigma* sp.1 | - |
| *Apterostigma* sp.2 | - |
| *Apterostigma* sp.3 | - |
| *Apterostigma* sp.4 | - |
| *Apterostigma urichii** Forel, 1893 | Forest Specialist |
| *Atta laevigata* (Smith, 1858) | Open Area Specialist |
| *Atta sexdens* (Linnaeus, 1758) | Generalist |
| *Atta* sp.1 | - |
| **Continue...** |  |
| *Atta* sp.2 | - |
| *Carebara* sp.1 | - |
| *Carebara* sp.2 | - |
| *Carebara* sp.3 | - |
| *Carebara* sp.4 | - |
| *Cephalotes atratus* (Linnaeus, 1758) | Generalist |
| *Cephalotes clypeatus* (Fabricius, 1804) | Generalist |
| *Cephalotes complanatus* (Guérin-Méneville, 1844) | Forest Specialist |
| *Cephalotes laminatus* (Smith, 1860) | Forest Specialist |
| *Cephalotes opacus* Santschi, 1920 | Forest Specialist |
| *Cephalotes pallidus** De Andrade, 1999 | Open-area Specialist |
| *Cephalotes pavonii* (Latreille, 1809) | Forest Specialist |
| *Cephalotes placidus* (Smith, 1860) | Generalist |
| *Cephalotes spinosus* (Mayr, 1862) | Forest Specialist |
| *Cephalotes umbraculatus* (Fabricius, 1804) | Generalist |
| *Crematogaster brasiliensis* Mayr, 1878 | Forest Specialist |
| *Crematogaster flavosensitiva* Longino, 2003 | Forest Specialist |
| *Crematogaster nigropilosa* Mayr, 1870 | Generalist |
| *Crematogaster* sp.1 | - |
| *Crematogaster* sp.2 | - |
| *Crematogaster* sp.3 | - |
| *Crematogaster* sp.4 | - |
| *Crematogaster* sp.5 | - |
| *Crematogaster* sp.6 | - |
| *Crematogaster* sp.7 | - |
| *Crematogaster* sp.8 | - |
| *Crematogaster* sp.9 | - |
| *Crematogaster* sp.10 | - |
| *Crematogaster* sp.11 | - |
| *Crematogaster* sp.12 | - |
| *Crematogaster* sp.13 | - |
| *Crematogaster* sp.14 | - |
| *Crematogaster* sp.15 | - |
| *Crematogaster* sp.16 | - |
| *Crematogaster* sp.17 | - |
| *Crematogaster* sp.18 | - |
| *Crematogaster* sp.19 | - |
| *Crematogaster* sp.20 | - |
| *Crematogaster* sp.21 | - |
| *Crematogaster* sp.22 | - |
| *Crematogaster* sp.23 | - |
| **Continue...** |  |
| *Crematogaster* sp.24 | - |
| *Crematogaster* sp.25 | - |
| *Crematogaster* sp.26 | - |
| *Crematogaster stollii* Forel, 1885 | Generalist |
| *Cyphomyrmex costatus** Mann, 1922 | Forest Specialist |
| *Cyphomyrmex hamulatus** Weber, 1938 | Forest Specialist |
| *Cyphomyrmex laevigatus* Weber, 1938 | Forest Specialist |
| *Cyphomyrmex rimosus* (Spinola, 1851) | Generalist |
| *Cyphomyrmex* sp.1 | - |
| *Cyphomyrmex* sp.2 | - |
| *Cyphomyrmex* sp.3 | - |
| *Cyphomyrmex* sp.4 | - |
| *Cyphomyrmex* sp.5 | - |
| *Cyphomyrmex* sp.6 | - |
| *Cyphomyrmex* sp.7 | - |
| *Hylomyrma blandiens* Kempf, 1961 | Forest Specialist |
| *Hylomyrma immanis* Kempf, 1973 | Forest Specialist |
| *Hylomyrma marielleae* Ulysséa, 2021 | Forest Specialist |
| *Lachnomyrmex pilosus** Weber, 1950 | Forest Specialist |
| *Leptogenys* sp.1 | - |
| *Megalomyrmex ayri* Brandão, 1990 | Forest Specialist |
| *Megalomyrmex drifti** Kempf, 1961 | Forest Specialist |
| *Megalomyrmex incisus** Smith, 1947 | Forest Specialist |
| *Megalomyrmex leoninus* Forel, 1885 | Forest Specialist |
| *Megalomyrmex* sp.n | - |
| *Megalomyrmex wallacei** Mann, 1916 | Forest Specialist |
| *Monomorium pharaonis* (Linnaeus, 1758) | Generalist |
| *Mycetomoellerius farinosus* (Emery, 1894) | Forest Specialist |
| *Mycetophylax faunulus* (Wheeler, 1925) | Forest Specialist |
| *Mycocepurus smithii* (Forel, 1893*)* | Generalist |
| *Myrmicocrypta* sp.1 | - |
| *Myrmicocrypta* sp.2 | - |
| *Myrmicocrypta* sp.3 | - |
| *Nesomyrmex* sp.n | - |
| *Ochetomyrmex neopolitus* Fernández, 2003 | Forest Specialist |
| *Ochetomyrmex semipolitus* Mayr, 1878 | Generalist |
| *Octostruma balzani* (Emery, 1894) | Forest Specialist |
| *Octostruma betschi* Perrault, 1988 | Forest Specialist |
| *Octostruma iheringi* (Emery, 1888) | Forest Specialist |
| *Octostruma obtusidens** Longino, 2013 | Forest Specialist |
| *Octostruma pexidorsum** Longino, 2013 | Forest Specialist |
| **Continue...** |  |
| *Octostruma* sp.1 | - |
| *Paratrachymyrmex mandibularis** (Weber, 1938) | Forest Specialist |
| *Paratrachymyrmex diversus* (Mann, 1916) |  |
| *Pheidole astur* Wilson, 2003 | Forest Specialist |
| *Pheidole bufo* Wilson, 2003 | Forest Specialist |
| *Pheidole cataractae* Wheeler, 1916 | Forest Specialist |
| *Pheidole fimbriata* Roger, 1863 | Forest Specialist |
| *Pheidole lancifera* Wilson, 2003 | Forest Specialist |
| *Pheidole leonina* Wilson, 2003 | Forest Specialist |
| *Pheidole lovejoyi* Wilson, 2003 | Forest Specialist |
| *Pheidole radoszkowskii* Mayr, 1884 | Generalist |
| *Pheidole sensitiva* Borgmeier, 1959 | Forest Specialist |
| *Pheidole* sp.2 | - |
| *Pheidole* sp.3 | - |
| *Pheidole* sp.4 | - |
| *Pheidole* sp.5 | - |
| *Pheidole* sp.6 | - |
| *Pheidole* sp.8 | - |
| *Pheidole* sp.10 | - |
| *Pheidole* sp.12 | - |
| *Pheidole* sp.13 | - |
| *Pheidole* sp.16 | - |
| *Pheidole* sp.17 | - |
| *Pheidole* sp.18 | - |
| *Pheidole* sp.19 | - |
| *Pheidole* sp.20 | - |
| *Pheidole* sp.21 | - |
| *Pheidole* sp.23 | - |
| *Pheidole* sp.25 | - |
| *Pheidole* sp.26 | - |
| *Pheidole* sp.28 | - |
| *Pheidole* sp.29 | - |
| *Pheidole* sp.31 | - |
| *Pheidole* sp.32 | - |
| *Pheidole* sp.33 | - |
| *Pheidole* sp.34 | - |
| *Pheidole* sp.35 | - |
| *Pheidole* sp.36 | - |
| *Pheidole* sp.38 | - |
| *Pheidole* sp.39 | - |
| *Pheidole* sp.40 | - |
| **Continue...** |  |
| *Pheidole* sp.41 | - |
| *Pheidole* sp.42 | - |
| *Pheidole* sp.43 | - |
| *Pheidole* sp.44 | - |
| *Pheidole* sp.45 | - |
| *Pheidole* sp.46 | - |
| *Pheidole* sp.47 | - |
| *Pheidole* sp.48 | - |
| *Pheidole* sp.49 | - |
| *Pheidole* sp.50 | - |
| *Pheidole* sp.51 | - |
| *Pheidole* sp.52 | - |
| *Pheidole* sp.53 | - |
| *Pheidole* sp.54 | - |
| *Pheidole* sp.55 | - |
| *Pheidole* sp.56 | - |
| *Pheidole* sp.57 | - |
| *Pheidole* sp.58 | - |
| *Pheidole* sp.60 | - |
| *Pheidole* sp.61 | - |
| *Pheidole* sp.62 | - |
| *Pheidole* sp.63 | - |
| *Pheidole* sp.64 | - |
| *Pheidole vorax* (Fabricius, 1804) | Forest Specialist |
| *Rogeria blanda** (Smith, 1858) | Forest Specialist |
| *Rogeria scobinata** Kugler, 1994 | Forest Specialist |
| *Rogeria* sp.1 | - |
| *Rogeria* sp.2 | - |
| *Sericomyrmex bondari* Borgmeier, 1937 | Forest Specialist |
| *Sericomyrmex mayri* Forel, 1912 | Generalist |
| *Sericomyrmex parvulus* Forel, 1912 | Generalist |
| *Sericomyrmex saussurei* Emery, 1894 | Forest Specialist |
| *Sericomyrmex* sp.1 | - |
| *Sericomyrmex* sp.2 | - |
| *Solenopsis geminata* (Fabricius, 1804) | Forest Specialist |
| *Solenopsis* gr. brevicornis | - |
| *Solenopsis* sp.1 | - |
| *Solenopsis* sp.2 | - |
| *Solenopsis* sp.3 | - |
| *Solenopsis* sp.4 | - |
| *Solenopsis* sp.5 | - |
| **Continue...** |  |
| *Solenopsis* sp.6 | - |
| *Solenopsis* sp.7 | - |
| *Solenopsis* sp.8 | - |
| *Solenopsis* sp.9 | - |
| *Solenopsis* sp.10 | - |
| *Solenopsis* sp.11 | - |
| *Solenopsis* sp.12 | - |
| *Solenopsis* sp.13 | - |
| *Solenopsis* sp.14 | - |
| *Solenopsis virulens* (Smith, 1858) | Forest Specialist |
| *Stegomyrmex connectens* Emery, 1912 | Forest Specialist |
| *Strumigenys beebei* (Wheeler, 1915) | Forest Specialist |
| *Strumigenys denticulata* Mayr, 1887 | Forest Specialist |
| *Strumigenys eggersi* Emery, 1890 | Generalist |
| *Strumigenys elongata* Roger, 1863 | Generalist |
| *Strumigenys godmani** Forel, 1899 | Forest Specialist |
| *Strumigenys* gr. hyphata sp.n | - |
| *Strumigenys gundlachi* (Roger, 1862) | Forest Specialist |
| *Strumigenys interfectiva* Lattke & Goitía, 1997 | Forest Specialist |
| *Strumigenys longispinosa* Brown, 1958 | Forest Specialist |
| *Strumigenys perparva** Brown, 1958 | Forest Specialist |
| *Strumigenys schulzi** Emery, 1894 | Forest Specialist |
| *Strumigenys trudifera* Kempf & Brown, 1969 | Forest Specialist |
| *Strumigenys urrhobia** (Bolton, 2000) | Forest Specialist |
| *Trachymyrmex* sp.1 | - |
| *Trachymyrmex* sp.2 | - |
| *Trachymyrmex* sp.3 | - |
| *Trachymyrmex* sp.4 | - |
| *Trachymyrmex* sp.5 | - |
| *Trachymyrmex* sp.6 | - |
| *Wasmannia auropunctata* (Roger, 1863) | Generalist |
| *Wasmannia iheringi** Forel, 1908 | Forest Specialist |
| *Wasmannia scrobifera** Kempf, 1961 | Forest Specialist |
| **Paraponerinae (1)** |  |
| *Paraponera clavata* (Fabricius, 1775) | Forest Specialist |
| **Ponerinae (45)** |  |
| *Anochetus diegensis* Forel, 1912 | Forest Specialist |
| *Anochetus mayri** Emery, 1884 | Forest Specialist |
| *Hypoponera* sp.1 | - |
| *Hypoponera* sp.2 | - |
| *Hypoponera* sp.3 | - |
| **Continue...** |  |
| *Hypoponera* sp.4 | - |
| *Hypoponera* sp.5 | - |
| *Hypoponera* sp.6 | - |
| *Hypoponera* sp.7 | - |
| *Hypoponera* sp.8 | - |
| *Hypoponera* sp.9 | - |
| *Hypoponera* sp.10 | - |
| *Hypoponera* sp.11 | - |
| *Hypoponera* sp.12 | - |
| *Hypoponera* sp.13 | - |
| *Hypoponera* sp.14 | - |
| *Hypoponera* sp.15 | - |
| *Hypoponera* sp.16 | - |
| *Hypoponera* sp.17 | - |
| *Hypoponera* sp.18 | - |
| *Hypoponera* sp.19 | - |
| *Mayaponera constricta* (Mayr, 1884) | Forest Specialist |
| *Mayaponera arhuaca* (Forel, 1901) | Forest Specialist |
| *Neoponera apicalis* (Latreille, 1802) | Forest Specialist |
| *Neoponera carinulata* (Roger, 1861) | Forest Specialist |
| *Neoponera globularia* (MacKay & MacKay, 2010) | Generalist |
| *Pachycondyla lenkoi** Kempf, 1962 | Forest Specialist |
| *Neoponera moesta* (Mayr, 1870) | Forest Specialist |
| *Neoponera* sp.1 | - |
| *Neoponera* sp.2 | - |
| *Neoponera oberthueri**(Emery, 1890) | Generalist |
| *Neoponera unidentata* (Mayr, 1862) | Forest Specialist |
| *Neoponera verenae* Forel, 1922 | Generalist |
| *Odontomachus bauri* Emery, 1892 | Generalist |
| *Odontomachus caelatus*Brown, 1976 | Forest Specialist |
| *Odontomachus haematodus* (Linnaeus, 1758) | Generalist |
| *Odontomachus meinerti* Forel, 1905 | Generalist |
| *Odontomachus* sp.1 | - |
| *Pachycondyla crassinoda* (Latreille, 1802) | Forest Specialist |
| *Pachycondyla harpax* (Fabricius, 1804) | Generalist |
| *Pachycondyla* sp.1 | - |
| *Platythyrea angusta* Forel, 1901 | Forest Specialist |
| *Rasopone* sp.1 | - |
| Thaumatomyrmex aff. atrox | - |
| **Proceratiinae (2)** |  |
| *Discothyrea neotropical** Bruch, 1919 | Forest Specialist |
| **Continue...** |  |
| *Probolomyrmex dentinodis** Oliveira & Feitosa, 2019 | Forest Specialist |
| **Pseudomyrmecinae (6)** |  |
| *Pseudomyrmex crudelis* Ward, 1999 | Forest Specialist |
| *Pseudomyrmex* sp.1 | - |
| *Pseudomyrmex* sp.2 | - |
| *Pseudomyrmex* sp.3 | - |
| *Pseudomyrmex* sp.4 | - |
| *Pseudomyrmex tenuis* (Fabricius, 1804) | Generalist |

**
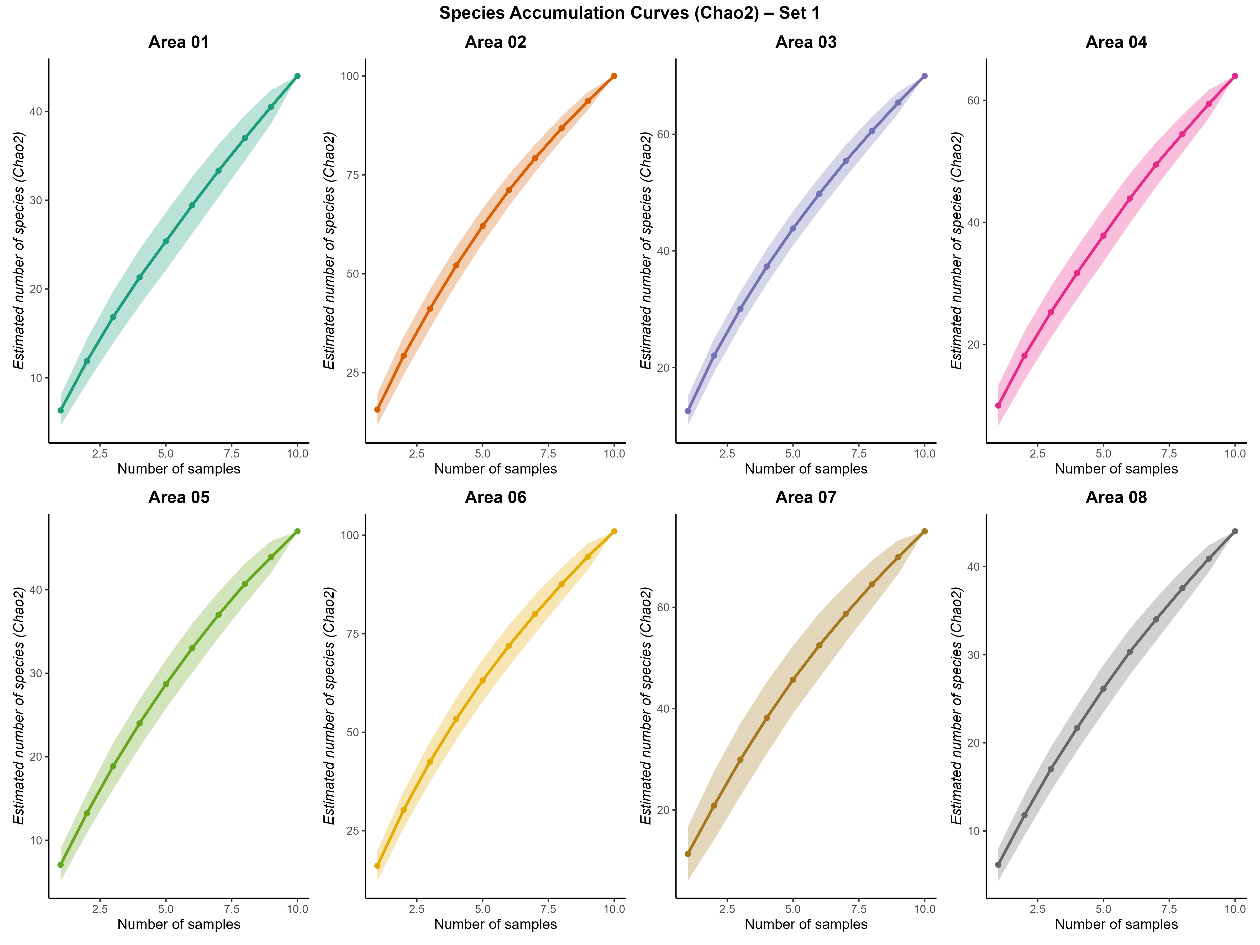
**

B

A

**
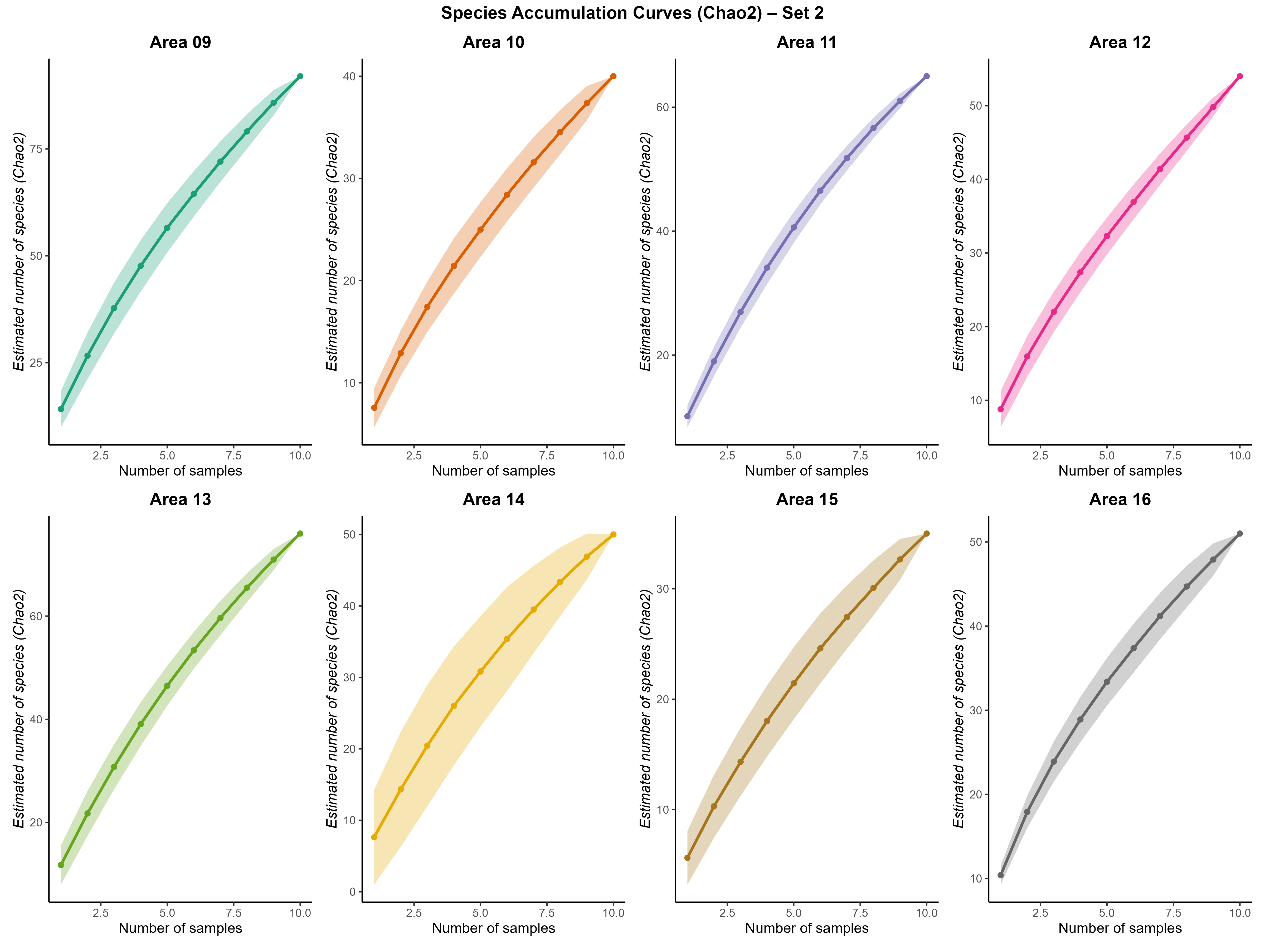
**

**Figure S1.** Ant species accumulation curves of the 16 sampling areas in Acre, Brazil, southwestern Brazilian Amazon. (A) Curves for the first eight sampling areas. (B) Curves for the remaining eight sampling areas.


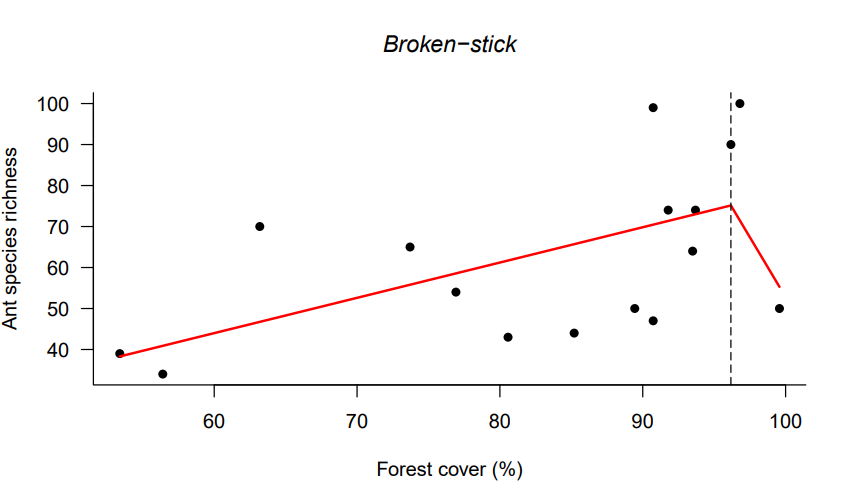

**Figure S2.** Broken stick model revealing the relationship between forest cover and richness of ant species (AIC: 146,923).


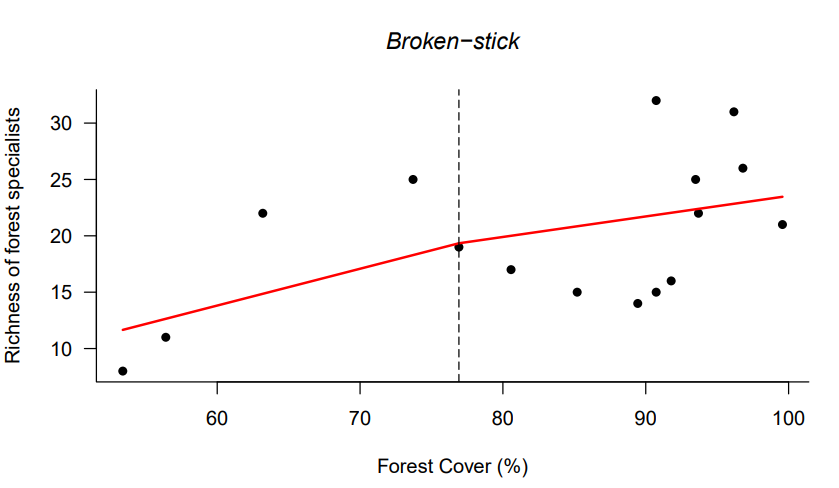


**Figure S3.** Broken stick model revealing the relationship between forest cover and richness of forest specialist ant species (AIC: 111,756).


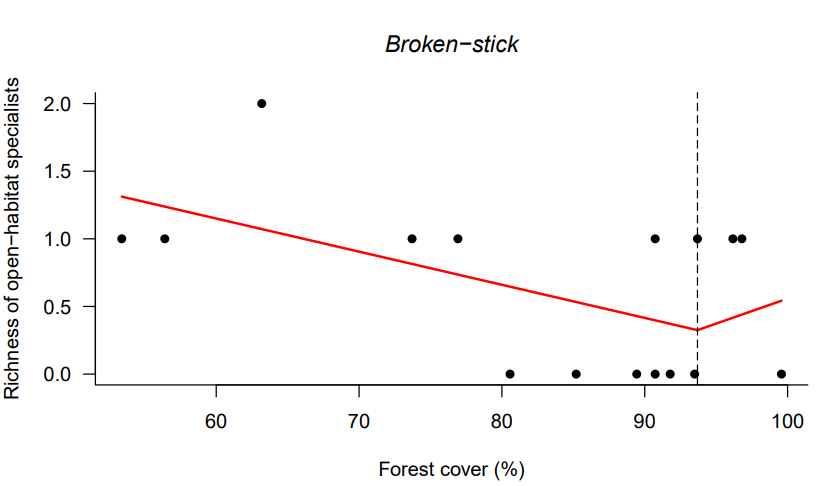


**Figure S4.** Broken stick model revealing the relationship between forest cover and richness of open area specialist ant species (AIC: 35,556).


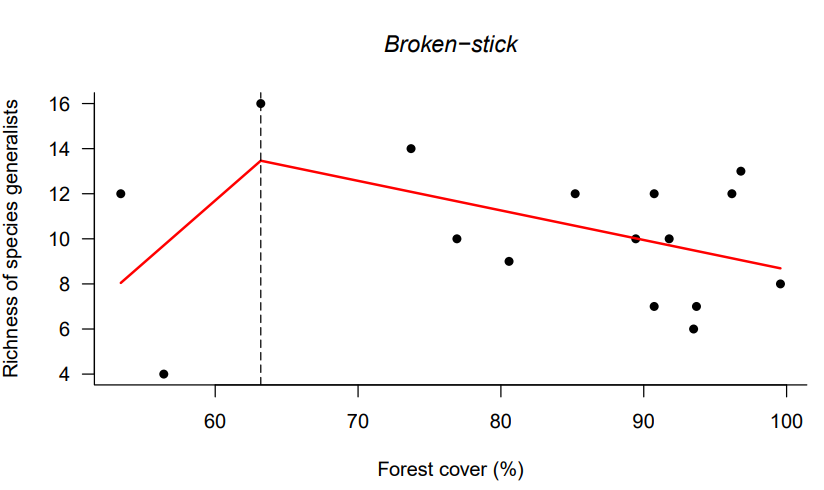


**Figure S5.** Broken stick model revealing the relationship between forest cover and richness of generalist ant species (AIC: 89,703).
